# Geographic Variation of the Incidence Rate of Lower Limb Amputation in Australia from 2007-12

**DOI:** 10.1371/journal.pone.0170705

**Published:** 2017-01-24

**Authors:** Michael P. Dillon, Lauren V. Fortington, Muhammad Akram, Bircan Erbas, Friedbert Kohler

**Affiliations:** 1 School of Allied Health, La Trobe University, Bundoora, Victoria, Australia; 2 Australian Collaboration for Research into Injury in Sports and its Prevention, Federation University, Ballarat, Victoria, Australia; 3 Australian Centre of Health and Social Research, Australian Catholic University, Melbourne, Victoria, Australia; 4 School of Psychology and Public Health, La Trobe University, Bundoora, Victoria, Australia; 5 Rehabilitation Medicine, Braeside Hospital, Prairiewood, New South Wales, Australia; 6 Faculty of Medicine, University of New South Wales, Kensington, New South Wales, Australia; Jichi Medical University, JAPAN

## Abstract

In Australia, little is known about how the incidence rate (IR) of lower limb amputation (LLA) varies across the country. While studies in other economically developed countries have shown considerable geographic variation in the IR-LLA, mostly these have not considered whether the effect of common risk factors are the same across regions. Mapping variation of the IR-LLA, and the effect of common risk factors, is an important first step to focus research into areas of greatest need and support the development of regional specific hypotheses for in-depth examination. The aim of this study was to describe the geographic variation in the IR-LLA across Australia and understand whether the effect of common risk factors was the same across regions. Using hospital episode data from the Australian National Hospital Morbidity database and Australian Bureau of Statistics, the all-cause crude and age-standardised IR-LLA in males and females were calculated for the nation and each state and territory. Generalised Linear Models were developed to understand which factors influenced geographic variation in the crude IR-LLA. While the crude and age-standardised IR-LLA in males and females were similar in most states and territories, they were higher in the Northern Territory. The effect of older age, being male and the presence of type 2 diabetes was associated with an increase of IR-LLA in most states and territories. In the Northern Territory, the younger age at amputation confounded the effect of sex and type 2 diabetes. There are likely to be many factors not included in this investigation, such as Indigenous status, that may explain part of the variation in the IR-LLA not captured in our models. Further research is needed to identify regional- and population- specific factors that could be modified to reduce the IR-LLA in all states and territories of Australia.

## Introduction

In Australia, there are some 8000 lower limb amputations (LLA) performed annually [1,2]. Despite a variety of targeted interventions, such as the increased availability of high-risk foot clinics and clear guidelines for their delivery[3], there has not been a reduction in the incidence rate of lower limb amputation (IR-LLA) at a national level [2].

While data describing the IR-LLA is available at a national level [2] and for isolated states[4,5], there are no studies that describe the IR-LLA for all Australian states and territories, and whether this has changed over time. Similarly, there are no studies that quantify whether the effect of common risk factors such as sex, age or the presence of diabetes are similar across different geographic regions.

Research in similar, economically-developed, countries has shown considerable geographic variation in the IR-LLA[6–10]. Synthesis of these investigations suggests that it is common for the IR-LLA to be two-three times higher in some regions compared to others. Such geographic variation in the IR-LLA may reflect that common risk factors for LLA are not equally distributed across the population, but rather geographically clustered. Researchers have suggested considerable variation in the geographic distribution of LLAs based on age, sex, diabetes, socioeconomic- and Indigenous- status, as well as remoteness [7,10–12]. Variations in healthcare between regions are also common [10] as exemplified by variation in the use of diagnostic angiography and revascularization procedures that are commonly performed to prevent amputation[13]. Unfortunately, no studies have examined whether the effect of common risk factors is the same across geographic regions.

Mapping the geographic variation of LLA and testing whether the effect of common risk factors is the same across regions, should be seen as an important first step to quantify the IR-LLA and understand what causes variation across Australia. A national analysis of geographic variation, including a breakdown by individual state and territory, is important to identify geographic regions where the IR-LLA is under- or over- represented. This approach is prudent because it enables future research to focus on regions of greatest need and to establish geographically relevant hypotheses for in-depth examination. In this way, researchers can examine the effect of a unique combination of risk factors specific to different geographic regions (e.g., the effect of Indigenous status may be important in the Northern Territory but not in other states or territories where the proportion of Indigenous Australians is small)[14]. Future studies may use these data as a reference point to compare changes over time and thereby establish whether interventions targeted to the needs of different geographic regions have been effective at reducing the IR-LLA.

Investigations describing the geographic variation in the IR-LLA in Australia are timely given the roll-out of major health reforms such as the National Disability Insurance Scheme (NDIS) and Health Workforce Australia (HWA). There is an opportunity to plan for the specialist services required by people facing LLA and to address the serious, long-term health and economic burden associated with LLA [11,15,16]. All parties involved in the provision and planning for these specialist preventative and rehabilitation services would be well served by data on the geographic variation of LLA that is currently not available.

The aim of this investigation was to describe the geographic variation in the IR-LLA across Australia and how this has changed over time. Given our hypothesis that there would be significant geographic variation in the IR-LLA across Australia, we also sought to understand the influence that sex, age, diabetes and time had on the relative risk (RR) of amputation in each region.

## Methods

### Ethics approval

Ethics approval for the project was granted by the South Australian Health Human Research Ethics Committee (HREC/14/SAH/96).

### Amputation data

Data were compiled by the AIHW from electronic, de-identified records for each episode of care held in the Australian National Hospital Morbidity database.[17] The Australian National Hospital Morbidity database has rigorous data collection procedures to ensure data accuracy. The National Health Data Dictionary[18] definitions form the basis of the database, ensuring a high standard of data comparability[17]. The AIHW undertakes extensive validations on receipt of data from state/territory health authorities and potential errors are queried with jurisdictions. Corrections and resubmissions may be made in response to these queries[19]. Additional drivers for appropriate coding and counting are the reimbursement system for surgical procedures and regular internal coding audits by each hospital. The reliability and accuracy of datasets for LLA procedures has been shown in two separate investigations[20,21]. While these observations do not exclude the possibility of some procedures being missed or miscoded, it does engender confidence that there is no systematic error of miscounting or miscoding.

All surgical procedures included in the database are coded according to the International Classification of Diseases 10th revision Australian Modification Australian Classification of Health Interventions (ICD-10-AM-ACHI) for the financial years 2007–8 to 20011–12[17]. The ICD-10-AM-ACHI is the national standard for procedure and intervention coding in Australian hospitals.

The amputation data describe each episode of care and as such, there is no ability to delineate between first and subsequent procedures on the same limb or to separate data by the number of individuals affected. All causes of amputation were included with the exception of auto-amputations, which are not recorded in the Australian National Hospital Morbidity database as these are not considered surgical procedures. Amputation procedures were categorised as either: transfemoral, transtibial, partial foot (excluding toes), toes and other; as detailed in S1 Appendix. Each episode of care also included information about geographic region, year of amputation, age group, sex, the presence of type 1 and type 2 diabetes as well as amputation level. Given that several states and territories (e.g., Northern Territory, Australian Capital Territory, Tasmania) had small sample sizes, it was necessary to limit the number of factors included in the model to ensure sufficient power and as such, only the most influential risk factors were included.

Geographic region was defined using the Australian Bureau of Statistics Australian Standard Geographical Classification (ASGC). The ASGC is the Australian standard for the collection and dissemination of geographic statistics and is a hierarchically structured classification with a number of increasingly smaller spatial units from: states/territories to statistical divisions and then statistical subdivision. For this investigation, we only reported data to the level of states/territories; here after referred to as geographic regions. These data cover, in aggregate, the whole of Australia without gaps or overlaps. Principal diagnoses for type 1 and type 2 diabetes were defined by the ICD-10-AM-ACHI codes E10.* or E11.*, respectively. Age was categorized in 5-year brackets up to 70–74 years after which people were grouped into a 75+ category.

Data were presented from the 1^st^ July to the 30^th^ June the following calendar year. As such the 2007–2008 period, as it is described in the database and this publication, includes data from the 1^st^ July 2007 to the 30^th^ June 2008.

### Population data

Data on Australia’s population were extracted from the Australian Demographics Statistics reports[22] which were downloaded as Microsoft Excel spreadsheets from the Australian Bureau of Statistics (ABS) website. Data were available for each year of the analysis including a breakdown by geographic region, sex and 5 year age group consistent with the aforementioned definitions.

### Analysis

The crude IR-LLA was given by the number of LLA in a geographic region divided by the total population of that geographic region and expressed per 100,000 population. The crude IR-LLA was determined for each year included in the analysis for both the nation as a whole as well as for each geographic region.

To enable comparisons between geographic regions without the confounding influence of different population structures, an age-standardised IR-LLA was calculated by the direct method[23] for both men and women using the Australian Standard Population (June 30, 2001)[24]. For each age-standardised IR-LLA in men and women, a 95% confidence interval (95% CI) was calculated assuming Poisson error.

To understand which factors influenced the geographic variation in the IR-LLA, a series of Generalized Linear Models (GLM) were developed assuming a quasi-Poisson distribution to account for the over dispersion. Data from each amputation were included in the model without aggregation. Time was considered to be a categorical variable in all analyses. We considered the crude IR-LLA for a geographic region as the dependent variable (as opposed to using age-standardised rates in men and women) so that age and sex could be considered as independent variables. The independent variables—age, sex, time, presence of type 1 diabetes and presence of type 2 diabetes–were entered as fixed effects. Sex and the presence of type 2 diabetes were considered as effect modifiers and as such, interaction terms were fitted and considered significant if the p-value of the estimated interaction term was less than 0.05.

To visualise the spatial structure in the data, choropleth maps were used to display the aggregated age-standardised IR-LLA for males and females[25]. In generating the maps, the IR-LLA were classified into five classes using an equal interval method.

Analyses were performed using Microsoft Excel (Microsoft, Redmond, Washington), R Statistical Software (R Foundation, Vienna, Austria) and QGIS 2.10 (QGIS Bonn, Germany).

## Results

### National sample characteristics

There were a total of 35,306 LLA performed in Australia between the 1^st^ July 2007 and 30^th^ June 2012. Almost three-quarters of these procedures were below-ankle procedures (Table 1). Toe amputations were the most common level accounting for more than 40% of the total number of LLA (Table 1). Partial foot amputations (excluding the toe level) were twice as common as transtibial amputations and nearly three times as common as transfemoral amputation (Table 1). Two thirds of the population undergoing LLA were aged over 60 years of age (Table 1). One-third of LLA occurred in people between 35–60 years, with a small proportion of people younger than 35 years (Table 1). Two-thirds of all LLA were performed for males (Table 1). Half of all LLA occurred in people with type 2 Diabetes Mellitus (Table 1).

**Table 1:** Demographic characteristics by state/territory for all years combined (from 2007–8 to 2011–12).

	Nationally	NSW	VIC	QLD	SA	WA	TAS	NT	ACT
All amputation procedures*	35306	10795	8581	7031	3481	3409	873	695	441
All amputation hospitalisations^	33050	10090	7998	6602	3302	3190	838	602	428
Level^#^									
Transfemoral	4100 (11.6)	1440 (13.3)	733 (8.5)	953 (13.6)	452 (13.0)	366 (10.7)	71 (8.1)	45 (6.5)	40 (9.1)
Transtibial	5524 (15.7)	1871 (17.3)	1156 (13.5)	1142 (16.2)	499 (14.3)	519 (15.2)	128 (14.7)	117 (16.8)	92 (20.9)
Partial foot (excl. toes)	10312 (29.2)	2779 (25.7)	2866 (33.4)	1874 (26.7)	1176 (33.8)	981 (28.8)	236 (27.0)	258 (37.1)	142 (32.2)
Toe(s)	15040 (42.6)	4633 (42.9)	3724 (43.4)	2991 (42.5)	1333 (38.3)	1507 (44.2)	420 (48.1)	270 (38.9)	162 (36.7)
Other~	330 (0.9)	72 (0.7)	102 (1.2)	71 (1.0)	21 (0.6)	36 (1.1)	18 (2.1)	5 (0.7)	5 (1.1)
Age^									
Mean (SD)	62.9 (15.3)	63.9 (14.9)	63.97 (15.1)	61.4 (16.0)	64.2 (14.0)	60.9 (16.0)	63.1 (14.9)	51.8 (14.3)	62.6 (15.8)
over 60 years	20524 (62.1)	6600 (65.4)	5300 (66.3)	3784 (57.3)	2136 (64.7)	1784 (55.9)	528 (63.0)	133 (22.1)	259 (60.5)
35–60 years	11026 (33.4)	3069 (30.4)	2378 (29.7)	2443 (37.0)	1063 (32.2)	1218 (38.2)	274 (32.7)	429 (71.3)	152 (35.5)
< 35 years	1500 (4.5)	421 (4.2)	320 (4.0)	375 (5.7)	103 (3.1)	188 (5.9)	36 (4.3)	40 (6.6)	17 (4.0)
Sex^									
Male	23583 (67.0)	6815 (67.5)	5292 (66.2)	4440 (67.3)	2231 (67.6)	2116 (66.3)	560 (66.8)	399 (66.3)	287 (67)
Female	11453 (33.0)	3275 (32.5)	2706 (33.8)	2162 (32.7)	1071 (32.4)	1074 (33.7)	278 (33.2)	203 (33.7)	141 (33)
Diabetes flag^									
Type 1	1596 (4.8)	469 (4.7)	378 (4.7)	360 (5.5)	126 (3.8)	146 (4.6)	62 (7.4)	18 (3.0)	37 (8.6)
Type 2	16387 (49.6)	4697 (46.6)	4089 (51.1)	3243 (49.1)	1646 (49.9)	1679 (52.6)	365 (43.6)	471 (78.2)	197 (46.0)

*number and % unless otherwise stated;—not delineated by state due to small numbers.

# based on total procedures at stated level, within the eight Australian States/Territories (n = 35016)

^ based on total hospitalisations (n = 33050).

~ other levels include ankle, hip and knee joint disarticulations.

### National incidence rate

Across the time series of this investigation, the crude IR-LLA was 32.4 per 100,000 population. The age-adjusted IR-LLA was twice as high in males (40.3 per 100,000 population; 95%CI 39.8–40.8) as it was in females (19.9 per 100,000 population; 95%CI 19.5–20.2) (Table 2).

**Table 2:** Age-standardised incidence rate for males and females by nation and state/territory per 100,000 person-years, from 2007–2008 to 2011–2012.

	n	Population^	Crude rate	Age-standardised incidence rate	95% confidence interval (age-standardised rate)	
*Females*						
National	11453	54795459	20.9	19.9	19.5	20.2
NSW	3459	17878177	19.4	17.7	17.2	18.4
VIC	2833	13647560	20.8	19.4	18.7	20.1
QLD	2267	10956561	20.7	21.1	20.2	22.0
SA	1113	4095158	27.2	22.4	21.1	23.8
WA	1116	5520373	20.2	20.6	19.4	21.8
TAS	286	1271217	22.5	19.2	17.0	21.6
NT	236	538384	43.8	59.3	50.6	69.6
ACT	143	888029	16.1	18.8	15.8	22.2
*Males*						
National	23853	54246578	44.0	40.3	39.8	40.8
NSW	7336	17572487	41.8	37.1	36.2	37.9
VIC	5748	13394775	42.9	38.9	37.9	39.9
QLD	4764	10931836	43.6	41.4	40.3	42.6
SA	2368	3998804	59.2	49.2	47.2	51.2
WA	2293	5647725	40.6	39.8	38.2	41.5
TAS	587	1241813	47.3	39.6	36.4	43.1
NT	459	583343	78.7	100.1	90.2	111.1
ACT	298	875795	34.0	38.6	34.3	43.3

^combined total population for the study period.

### Factors influencing the national incidence rate

A number of factors were significant influences on the crude IR-LLA at a national level. Being male (RR 1.25; 95%CI 1.13–1.37, p<0.001) significantly increased the RR of LLA. There was a significant interaction between sex and type 2 diabetes. Holding all other variables constant, the presence of type 2 diabetes increased the RR more in females (RR 1.73; 95%CI 1.55–1.93) than in males (RR 1.32; 95%CI 1.04–1.67). Age was not a significant influence on RR of LLA (RR 1.00; 95%CI 0.998–1.003, p = 0.70). With respect to the baseline year of 2007–08, the RR did not change significantly over time, except for the 2009–10 year, in which the RR declined 11% (RR 0.89; 95%CI 0.81–0.97, p = 0.01).

### Region-by-region sample characteristics

While people undergoing LLA were similar across most geographic regions, people in the Northern Territory were typically younger and a larger proportion had type 2 Diabetes (Table 1). The proportion of procedures performed at the different amputation levels was fairly consistent in all regions (Table 1).

### Region-by-region incidence rate

**Fig 1:**
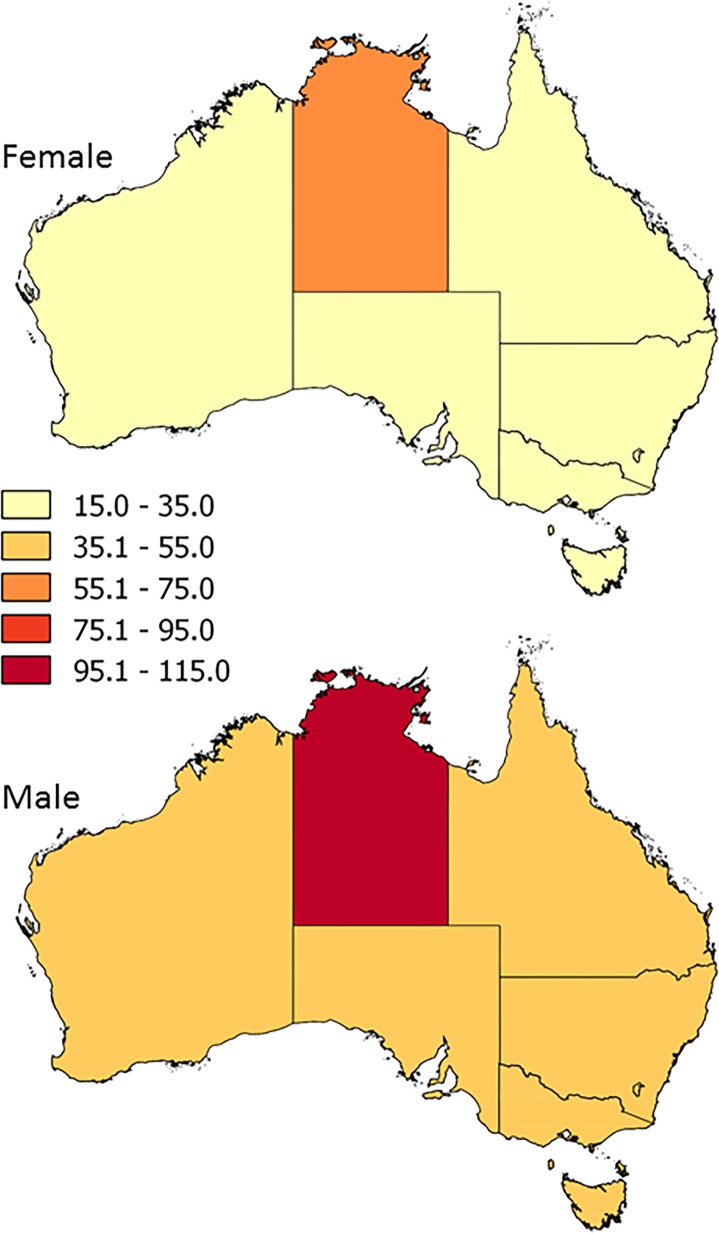
Geographic variation in the age-adjusted incidence rate of lower limb amputation for females (top) and males (bottom) across Australia from 2007–8 to 2011–12. Incidence rates expressed per 100,000 population by state/territory.

While the age-standardised IR-LLA for females (approx. 20 per 100,000 population) and males (approx. 40 per 100,000 population) were similar between regions, the respective rates were both higher in the Northern Territory (Fig 1, Table 2).

### Factors influencing region-by-region incidence rate

While age had a significant influence on the crude IR-LLA in all regions, such that increased age was associated with an increase in the crude IR-LLA (Table 3).

**Table 3:** Relative risk and 95%CI (in brackets) describing the influence of the independent variables on the crude incidence rate of lower limb amputation. The relative risk for each year is expressed relative to the baseline year 2007–8.

	NSW	VIC	QLD	SA	WA	TAS	NT	ACT
Intercept	0.37 (0.32–0.42)	0.48 (0.42–0.55)	0.49 (0.43–0.56)	1.70 (1.49–1.94)	0.91 (0.80–1.05)	5.43 (4.73–6.21)	2.36 (1.82–3.04)	4.92 (3.92–6.14)
Age	1.01 (1.003–1.007)	1.01 (1.003–1.007)	1.01 (1.008–1.012)	1.00 (1.000–1.004)	1.01 (1.010–1.013)	1.00 (1.001–1.005)	1.05 (1.047–1.055)	1.016 (1.013–1.019)
Sex (male)	1.21 (1.14–1.28)	1.25 (1.18–1.33)	1.16 (1.10–1.22)	1.25 (1.19–1.32)	1.19 (1.13–1.26)	1.24 (1.17–1.31)	0.90 (0.83–0.98)	1.32 (1.22–1.43)
Type 2 Diabetes	1.11 (1.05–1.17)	1.11 (1.05–1.17)	1.09 (1.04–1.15)	1.10 (1.05–1.15)	1.06 (1.01–1.12)	1.02 (0.96–1.07)	0.86 (0.78–0.95)	1.05 (0.97–1.13)
Type 1 Diabetes	1.01 (0.88–1.15)	1.07 (0.94–1.20)	1.03 (0.91–1.16)	1.06 (0.94–1.20)	1.00 (0.87–1.14)	0.97 (0.88–1.08)	0.90 (0.64–1.24)	0.90 (0.77–1.05)
Time, 2008–09	0.96 (0.88–1.04)	0.97 (0.89–1.04)	0.97 (0.90–1.05)	0.97 (0.91–1.05)	0.97 (0.89–1.04)	0.98 (0.91–1.06)	0.87 (0.77–0.99)	0.85 (0.76–0.96)
Time, 2009–10	0.94 (0.87–1.02)	0.93 (0.86–1.00)	0.93 (0.86–1.00)	0.97 (0.90–1.04)	0.90 (0.83–0.98)	0.96 (0.88–1.04)	0.86 (0.75–0.98)	0.81 (0.71–0.92)
Time, 2010–11	0.93 (0.86–1.01)	0.94 (0.87–1.02)	0.92 (0.85–0.99)	0.92 (0.85–0.99)	0.88 (0.82–0.96)	0.95 (0.88–1.02)	0.79 (0.70–0.90)	0.88 (0.79–0.99)
Time, 2011–12	0.92 (0.85–1.00)	0.88 (0.82–0.96)	0.90 (0.84–0.97)	0.95 (0.88–1.02)	0.87 (0.81–0.95)	0.89 (0.82–0.97)	0.74 (0.66–0.84)	0.79 (0.70–0.89)

Sex was a significant influence on the crude IR-LLA in all regions. In most regions, being male increased the RR by between 15–32% (Table 3). By contrast, in the Northern Territory being male reduced the RR by 10% (RR 0.90, 95%CI 0.83–0.98).

The influence of type 2 diabetes varied between regions (Table 3). Type 2 diabetes significantly increased the RR between 6–11% in Western Australia, South Australia, Queensland, New South Wales and Victoria. Type 2 diabetes was not a significant influence in the crude IR-LLA in the Australian Capital Territory (RR 1.05, 95%CI 0.97–1.13) or Tasmania (RR 1.02, 95%CI 0.96–1.07). Only in the Northern Territory was type 2 diabetes associated with a reduced RR (RR 0.86, 95%CI 0.78–0.95).

Given these observations it seems reasonable to contend that age may have confounded the effect that sex and type 2 diabetes had on the crude IR-LLA. In most regions, the crude and age-standardised IR-LLA were similar indicating that the influence of age-standardisation was small (Table 2). By comparison, the age-standardised IR-LLA was about 30% larger than the crude IR-LLA in the Northern Territory, suggesting that the influence of age was disproportionately large in both men and women (Table 2). To test if age was an effect modifier of type 2 diabetes and sex on the crude IR-LLA in the Northern Territory, data were stratified into three age groups. Holding all other variables constant, the RR associated with type 2 diabetes did not have a significant effect on the crude IR-LLA in the Northern Territory when stratified by age—(age<35: RR 1.00, 95%CI 0.84–1.19, p = 0.98; age35 to 60: RR 1.09, 95%CI 0.96–1.24, p = 0.20; age >60: RR 0.96, 95%CI 0.80–1.16, p = 0.66). The RR associated with sex (being male) was significant for the 35–60 year age group, but not the other age groups (age<35: RR 0.97, 95%CI 0.83–1.13; age 35–60: RR 0.83, 95%CI 0.76–0.91; age>60: 0.86, 95%CI 0.72–1.04). To confirm the confounding effect of age on sex and type 2 diabetes in the Northern Territory, separate models were developed by first including and then excluding age. When age was included in the model, the effect of sex (RR 0.90, 95%CI 0.83–0.98) and type 2 diabetes (RR 0.86, 95%CI 0.78–0.95) in the Northern Territory was significant. When age was removed from the model, neither sex (RR 0.96, 95%CI 0.83–1.12) nor type 2 diabetes (RR 0.95, 95%CI 0.79–1.14) had a significant influence on the RR in the Northern Territory.

The effect of time varied between regions. With reference to the baseline year of 2007–8, the RR of LLA reduced each year in Western Australia, Northern Territory and the Australian Capital Territory (Table 3). In other regions, reductions in the RR since 2007–8 were either not significant or varied over time (Table 3).

## Discussion

### Do incidence rates very between geographic regions?

In response to our original research question, both the all-cause, crude and age-standardised IR-LLA in males and females were very similar across geographic regions except for the Northern Territory.

A number of studies have described much larger variation in the IR-LLA (3–10 fold) than was observed in this study [6–9,26,27]. While most of these studies were not designed to test whether factors had the same effect across geographic regions [6–8,26,27], and thereby evidence the underlying cause of such variation in the IR-LLA, the available descriptive data suggests that the IR-LLA varied more between geographic regions in cohorts with diabetes than without [6,27] and more in female cohorts than in males[27]. Using descriptive data presented by van Houtum et al. [27] and our own application of simple linear regression, we suggest that part of the variation in the IR-LLA between regions could be explained by the differing proportions of people with diabetes in those regions (r^2^ = 0.44; F_1,25_ = 19.84, p<0.001). There is also some evidence to suggest that variation in the IR-LLA between regions may be similar for cohorts with amputation below or above the ankle [8,26].

Investigators have suggested many other sources of geographic variation, with varying degrees of rigor. For example, it has been suggested that the IR-LLA may very between regions because at-risk populations tend to be clustered given socioeconomic factors[28] or because the decision to amputate is highly variable, unlike more straightforward treatment decisions such as internal fixation of a fractured hip [6,9].

### Which factors influenced the RR of amputation and did their influence differ across geographic regions?

While age, sex, type 2 diabetes and time all had a significant effect on RR-LLA, their influence varied across regions. For example, a diagnostic-flag for type 2 diabetes was associated with an increase the RR-LLA in five regions, had no effect in the Australian Capital Territory or Tasmania and was associated with a reduction in the RR-LLA in the Northern Territory. Similarly, being male was associated with an increased RR-LLA in most regions, but not in the Northern Territory.

There were complex interactions between these variables. For example, age confounded the effect of sex and type 2 diabetes in the Northern Territory. When the confounding effect of age was controlled for, the RR-LLA in the Northern Territory was not significantly increased by being male and having type 2 diabetes.

Given that the influence of these factors varied across regions, it is important that regional specific hypotheses can be developed to better understand the RR-LLA.

### Are there other factors that might influence the RR of amputation?

A number of factors not included in our investigation may influence the IR-LLA including: race, renal disease, hypertension, congestive heart failure, dementia, chronic obstructive pulmonary disease; as illustrative examples.[7] Previous investigations have highlighted that the IR-LLA is often disproportionately high in regions with large proportions of Indigenous people[12] and this may, in part, explain the higher IR-LLA in the Northern Territory compared to other regions. In the Northern Territory, one-third of the population are estimated to be of Indigenous origin[14]. By contrast, less than 4% of the population of all other states and territories are estimated to be of Indigenous origin[14].

It is likely that being an Indigenous Australian is a proxy for various determinants of health that, in turn, impact the risk of amputation[29,30]. For example, Aboriginal and Torres Strait Islander people have higher prevalence of type 2 diabetes, circulatory disease and end stage renal disease compared to other Australians [31] and there are known associations between these conditions and the risk of ulceration and subsequent amputation[32,33].

Understanding the unique populations in different geographic regions is key to developing regional specific hypotheses that can inform future work aimed at reducing the RR-LLA in each region of Australia.

We were unable to identify other studies that evaluated the effect of age, sex, diabetes or time (or any other factors known to influence the IR-LLA) in different geographic regions. Results from this investigation show that there may be important regional differences in the influence of these factors. For example, in this study, type 2 diabetes did not have a significant influence on the RR-LLA in Tasmania or the Australian Capital Territory which may suggest something unique about these regions or that the relatively small number of amputations and small population in these regions masked the true effect.

### Limitations

This investigation sought to describe geographic variation in the IR-LLA across Australia for the first time and to understand the effect that the most common risk factors (i.e., sex, age, diabetes and time) have on the RR of LLA in each region. To accomplish these aims we used a large national dataset and purposefully included all levels- and causes of- amputation and used the Australia population as our denominator in calculating the IR-LLA. There are a number of limitations associated with this approach.

Each case reflects a hospitalisation with one or more surgical procedures, not an individual person, which artificially inflates the number of people affected by about 25% given the incidence of repeat amputations[34,35]. As such, the IR-LLA reported in this study will be overestimated relative to other investigations that report only index (or first ever) amputation for a given individual[36,37].

Even though the dataset includes more than 35,000 LLAs from 2007–8 to 2011–12, the numbers of cases are relatively small in some geographic regions, notably the Northern Territory, Tasmania and the Australian Capital Territory. This is even more so when considered by sex or 5-year age group. As a result, the 95% confidence intervals around the IR-LLA are large in these geographic regions and our ability to measure the true effect of some factors may be limited given there may not be sufficient power to detect strata-specific associations.

A small proportion of amputation procedures (< 1%) were not attributable to one of the geographic regions (e.g., no fixed address). While these were counted in the national totals, they were unable to be included in analyses by geographic region and as such, there are small discrepancies in the figures reported.

We limited our choice of independent variables to key factors (i.e., age, sex, type 1 diabetes, type 2 diabetes, time) that have been shown to influence the RR of LLA across geographic regions. While we had hoped to include Indigenous status among a number of other factors in our analysis, we had to be pragmatic about our choice of independent variables given the AIHW’s policies to protect the privacy of individuals. We hope that future investigators can develop geographic specific hypotheses given an understanding of the regions of particular interest and use the insights from this investigation to inform their choice of independent variables that matter most. For example, investigators may consider requesting data from the AIHW by 10-year age group. The resulting age- and geographic-strata may then be large enough to protect the privacy of individuals, even if Indigenous Status were included. Given the small samples in some geographic regions, particularly when considered by statistical division, sex and 5-year age group, researchers will only be able to include a small handful of factors in their models and maintain statistical power. Acknowledging the need to thoughtfully select variables for inclusion in the model, there are additional patient related risk factors that would likely enhance future work such as data describing the presence of renal disease.

Interpreting the data for the Northern Territory presented some specific challenges and there are limitations to what we can infer from the IR-LLA in the Northern Territory given the higher proportion of Aboriginal and Torres Strait Islander people compared to other regions. We were unable to obtain information about Indigenous status in this dataset with the required level of detail for age group, sex and geographic region given policies to protect privacy. There are also likely to be other factors that interact to influence the unique findings in the Northern Territory that were not modelled in this investigation including the tendency for later diagnosis of type 2 diabetes in the Aboriginal and Torres Strait Islander people or diagnosis only when complications have developed, a higher rate of hospitalisation admissions for complications of diabetes and a higher rate of mortality at a younger age compared to non-Indigenous people.[38–40]

### Further research

Future research can build on results from this study, focusing on smaller geographic regions (e.g., statistical divisions within states or territories), specific at-risk populations (e.g., Indigenous Australians, those with diabetes) as well as determining the effect that other factors (e.g., social deprivation, remoteness) have on the IR-LLA. Research in the UK has followed this sequenced approach where considerable variation within smaller geographic regions was identified [26] following an earlier preliminary work using a national population-based study[8].

This preliminary work indicates that types of amputations may vary significantly between geographic regions. As such, there is a strong justification for further work that presents IR data stratified by amputation level for each geographic region. This preliminary work suggests there is a need for separate regression models based on toe, partial foot (excluding toe) as well as transtibial and transfemoral amputation rather than more common categorizations (e.g., major or minor; below- or above-ankle).

Analyses by smaller geographic regions within states/territories (e.g., statistical divisions or subdivisions) would be a valuable next step. These analyses should consider use of spatial epidemiological methods given that controlling for the similarity of outcomes in nearby regions has been shown to influence the significance of some factors that contribute to geographic variation^9^.

## Conclusion

While the crude and age-standardised IR-LLA in males and females were similar in most states and territories, they were higher in the Northern Territory. The effect of advancing age, being male and the presence of type 2 diabetes was associated with an increase of RR-LLA in most regions. There were complex interactions between these factors in some regions. For example, the younger age at amputation confounded the effect of sex and type 2 diabetes in the Northern Territory. There are also likely to be factors not considered as part of this investigation, such as the large proportion of Aboriginal and Torres Strait Islander people in the Northern Territory, which may explain part of the variance in the IR-LLA across Australia. Further research is needed to understand the complex population- and regional- specific factors that influence the RR-LLA so that we may continue to reduce the IR-LLA across Australia.

## Supporting Information

S1 Appendix International Classification of Disease (ICD-10-AM-ACHI) codes identifying lower limb amputation procedures, descriptors and how they were categorized in this investigation. (DOCX)
