# Supplementary material for: Geographic Variation of the Incidence Rate of Lower Limb Amputation in Australia from 2007-12
Source: PLoS One. 2017 Jan 24;12(1):e0170705. doi: 10.1371/journal.pone.0170705 (PMC5261737; doi:10.1371/journal.pone.0170705)
Supplement: S1 Appendix — (DOCX) [file pone.0170705.s001.docx]

**S1 Appendix.** International Classification of Disease (ICD-10-AM-ACHI) codes identifying lower limb amputation procedures, descriptors and how they were categorized in this investigation.

| **ICD-10-AM-ACHI** | **Descriptor** | **Categorisation** |
| --- | --- | --- |
| 44373-00 | Hindquarter / hemipelvectomy amputation | Other |
| 44370-00 | Amputation at hip | Other |
| 44367-00 | Amputation above knee | Transfemoral |
| 44367-01 | Disarticulation at knee | Other |
| 44367-02 | Amputation below knee | Transtibial |
| 44361-01 | Amputation of ankle through malleoli of tibia and fibula | Other |
| 44361-00 | Disarticulation through ankle | Other |
| 44364-00 | Midtarsal amputation | Partial foot (excl. toes) |
| 44364-01 | Transmetatarsal amputation | Partial foot (excl. toes) |
| 44358-00 | Amputation of toe including metatarsal bone | Partial foot (excl. toes) |
| 44338-00 | Amputation of toe | Toe(s) |
| 90557-00 | Disarticulation through toe | Toe(s) |
